# Case report: Chronic neutrophilic leukemia associated with monoclonal gammopathies. A case series and review of genetic characteristics and practical management

**DOI:** 10.3389/fonc.2022.1014671

**Published:** 2022-12-07

**Authors:** Gaël Vermeersch, Michel Delforge, Violaine Havelange, Carlos Graux, Lucienne Michaux, Timothy Devos

**Affiliations:** ^1^ Department of Hematology, University Hospitals Leuven, KU Leuven, Leuven, Belgium; ^2^ Department of Hematology, Université Catholique de Louvain Saint-Luc, Woluwe-Saint-Lambert, Belgium; ^3^ Department of Hematology, Université Catholique de Louvain, CHU UCL Namur - site Godinne, Yvoir, Belgium; ^4^ Center for Human Genetics, KU Leuven, Leuven, Belgium; ^5^ Department of Microbiology and Immunology, Laboratory of Molecular Immunology (Rega Institute), KU Leuven, Leuven, Belgium

**Keywords:** myeloproliferative disorders, chronic neutrophilic leukemia, monoclonal gammopathy, multiple myeloma, myeloid malignancy, myeloproliferative neoplasm

## Abstract

Chronic neutrophilic leukemia (CNL) is a rare but potentially aggressive *BCR::ABL1* negative myeloproliferative neoplasm, characterized by sustained mature, neutrophilic leukocytosis. The discovery of key driver mutations in the colony-stimulating-factor-3 receptor (*CSF3R*) gene resulted in the updated World Health Organization (WHO) diagnostic criteria in 2016. A significant number of CNL cases have been associated with plasma cell dyscrasias, predominantly multiple myeloma (MM) and monoclonal gammopathy of unknown significance (MGUS). Compared to pure CNL, mutated *CSF3R* is infrequently reported in CNL cases associated with monoclonal gammopathies (MG). Until now it remains unclear whether CNL and occurring plasma cell neoplasms are clonally related or CNL is developing secondary to the underlying dyscrasia. Owing to its rarity, currently no standard of care management exists for CNL and MG-associated CNL. In this case series we report the multi-center experience of five MG-associated CNL cases with a median age of diagnosis of 69 years. Three patients (66%) showed predominance of lambda light chain expression. Four (80%) eventually evolved to MM, and one CNL-MGUS patient developed secondary acute myeloid leukemia (AML). Mutated *CSF3R* was present in the patient who developed AML but was absent in other cases. To assess possible associated genetic aberrations we performed recurrent analysis with next-generation sequencing (NGS). Two patients (40%) deceased with a median time of survival of 8 years after CNL diagnosis. Three (60%) are currently in follow-up with no reoccurring leukocytosis. This case series, followed by a short review, provides a long-term clinical and genetic overview of five CNL cases associated with MG.

## Introduction

Chronic neutrophilic leukemia (CNL) is an infrequent *BCR::ABL1* negative myeloproliferative neoplasm (MPN) defined by sustained, mature neutrophilia and bone marrow hypercellularity with granulocytic hyperplasia (1, 2). Approximately 200 CNL cases have been described; however, many of these do not meet the current diagnostic criteria (3). The identification of key driver mutations in the colony-stimulating-factor-3 receptor (*CSF3R*) gene resulted in the updated diagnostic criteria by the World Health Organization (WHO) in 2016 (4, 5). *CSF3R* is mutated in up to 80% of CNL cases (6). Approximately 32% of CNL cases are associated with plasma cell dyscrasias, wherein *CSF3R* tends to be less frequently mutated (1, 7). It remains unclear whether both entities should be considered clonally related or neutrophilia is provoked by the plasma cell dyscrasia (1). The majority of CNL-associated paraproteinemias express lambda light chains (8). The lack of genetic markers and arbitrary WHO-diagnostic criteria, such as leukocyte count, challenges the diagnosis. This case series provides a long-term clinical and genetic overview of five CNL cases associated with monoclonal gammopathies (MG). Four patients developed multiple myeloma (MM), and one patient developed secondary acute myeloid leukemia (AML).

## Case description

### Case 1

In 2012, a 61-year-old woman was referred due to persistent, incremental neutrophilia since 2009. Medical history included no major abnormalities. At presentation medical therapy comprised estriol cream and alprazolam. She had one sibling diagnosed with polycythemia vera (PV). Ultrasound excluded hepatosplenomegaly. Serum electrophoresis and immunofixation identified immunoglobulin G (IgG) kappa-type paraproteinemia (cfr. **Table 1** for results of the full blood analysis from all patients). Bone marrow biopsy showed hypercellularity without dysplasia. Karyotyping resulted normal. Positron emission tomography-computed tomography (PET-CT) showed no bone lesions. No therapy was initiated. In 2016, MGUS evolved to MM “Revised International Staging System” stadium I (R-ISS). M-peak rose from 10 to 32 g/L. Bone marrow biopsy showed right-shifted hypercellularity (90%) with approximately 73.3% segmented neutrophils and central plasmacytosis of 35% without central blast excess. Amyloidosis was excluded by Congo Red staining. Next-generation sequencing (NGS) and fluorescence *in situ* hybridization (FISH) resulted normal. Initiation of bortezomib-thalidomide-dexamethasone (VTD) resulted in a very good partial response (VGPR) after six cycles. Autologous hematopoietic stem cell transplantation (HSCT) was executed in 2017. Six months later, MM progressed and FISH revealed a mono-allelic loss of 17p13/*TP53* (February 2018). Daratumumab-lenalidomide-dexamethasone (DRd) was started as the patient showed intolerance towards carfilzomib-lenalidomide-dexamethasone (KRd). Six months afterwards, fourth-line therapy with bendamustine was started as PET-CT showed disease progression. The patient died due to progressive multiple myeloma, respectively 9 and 3 years after CNL and MM diagnosis. No reoccurrence of peripheral blood leukocytosis ≥25 × 10^9^/L was observed after the initiation of VTD and following therapies.

**Table 1:** Overview of clinical and laboratory findings at presentation and follow-up.

	Patient 1 (F)		Patient 2 (M)			Patient 3 (F)		Reference range	Patient 4 (M)				Reference range	Patient 5 (F)			Reference range
	2012	2016	2013	2016	2020	Jul/2018	Nov/2018		Mar/2019	May/2019	Jun/20	Aug/21		Jan/20	Jul/20	Oct/20	
**Stage of disease**	CNL+MGUS	CNM+MM	CNL+DLBCL+MGUS	CNL+MGUS	CNL+MM	CNL	CNL+MM		CNL	CNL+AML	Relapse AML	1 y post HSCT		CNL+smoldering MM	CNL+MM	CNL+MM	
**Age (years)**	61	65	74	77	81	73	73		69	69	70	71		66	66	67	
**B-symptoms**	Yes	No	No	No	No	Yes	No		No	No	No	No		Yes	No	No	
**Hepatosplenomegaly** **(ultrasound)**	No	No	No	No	No	Yes	No		No	No	No	No		No	No	No	
**Treatment**	/	VTD, KRd, DRd, bendamustine	R-CHOP, R, intrathecal MTX, ARA-C&HCT	/	/	/	VMP		/	/	DAC+HSCT	/		/	VRD	VRD cycle 3	
**YoS after diagnosis**	9 y	3 y	7 y	4 y	<1 month	>4 y	>4 y		>3 y	>3 y	>3 y	>1 y		>2 y	>2 y	>1 y	
**Current status**	Deceased		Deceased			Alive			Alive					Alive			
**Laboratory findings**																	
Hb (g/L)	122	101	116	109	70	109	93	120–160	116	83	88	109	133–176	104	132	132	122–150
RBC count (10^12/L)	4.03	3.16	3.72	3.49	2.3	3.52	2.82	3.9–5.6	3.41	2.47	2.58	3.16	4.10–5.70	2.95	4.37	4.29	4.0–6.0
MCV (fL)	94.8	98.4	93.3	93.4	91.3	96.2	104.3	76.0–96.0	100.3	99.6	103.5	106.0	80.1–99.8	105.8	93.6	94.4	85.0–95.0
WBC count (10^9/L)	41.32	17.71	43.69	53.14	7.98	65.2	60.85	4.0–10.0	13.62	18.54	1.57	4.15	3.70–9.50	41.54	34.15	11.63	4.0–10.0
Neutrophils %	87.8	74.7	90	91.3	73	89.1	88	38.0–77.0	70.1	74.6	19.5	47.8	45.0–70.0	87.5	80.7	67.6	40.0–70.0
Eosinophils %	0.3	0.6	0.3	0.3	1	0	0	≤6.0	3.1	2	0	1.7	1.0–6.0	0	0.5	1.8	0.5–6.0
Basophils %	0.3	0.1	0.3	0.1	0	0	0	≤1.0	0.8	3.3	0.7	0.7	0.0–2.0	0	0.4	0.4	0.1–2.0
Lymphocytes %	8.2	19.5	5.2	5.7	19	3.2	5	20–50	19.7	10.7	75.8	36.1	20.0–45.0	3.6	10.1	16.7	20.0–50.0
Monocytes%	3.5	5.1	3.6	2.6	3	4.5	7	2.0–10.0	6.3	2.7	3.3	<1.0	2.0–12.0	2.2	4.1	11.1	5.0–10.0
Blasts %	/	/	/	/	/	/	/	/	<1.0	2.7	<1.0	<1.0	<1.0	/	/	/	/
eGFR (CKD-EPI mL/min/1.73m^2^)	>90	101	27	27	17	46	60	/	94	108	133.72	71.56	/	59	82	63	/
LDH (U/L)	160	163	/	139	247	260	232	135–250	/	198	124	201	120–246	261	177	202	<250
Vitamin B12 (ng/L)	>2000	/	/	/	196	>2000	/	191–663	/	928	/	/	239–931	>2000	/	/	196–729
M-spike (g/L)	10.1	32.1	/	/	/	/	/	/	/	/	/	/	/	9.6	14.23	5.5	/
IgG (g/L)	18.4	42	/	2.13	2.19	/	3.87	7.51–15.60	/	2.53	/	7.00	0.88–4.10	15.2	16.61	10.52	7.0–16.0
IgA (g/L)	0.6	0.16	/	13.5	36.6	/	0.71	0.82–4.53	/	11.97	/	0.16	6.90–14.00	0.71	0.66	0.59	0.7–0.4
IgM (g/L)	0.57	0.19	/	0.05	<0.04	/	0.10	0.46–3.04	/	1.12	/	0.06	0.34–2.10	0.57	0.55	0.44	0.4–2.3
Serum k/λ	1.37	/	/	/	1.306	/	0.066	0.399–0.984	/	/	/	/	0.399–0.984	/	/	1.29	0.399–0.984
Urine k/λ	/	/	/	Nc	Nc	/	Nc	0.7–6.2	/	/	/	/	0.7–6.2	/	/	/	0.7–6.2
**Blood film**	Left-shifted, TG, DB	/	Left-shifted, TG	/	TG	Left-shifted, TG,DB	TG,DB		/	/	/	/		/	/	/	

AML, acute myeloid leukemia; ARA-C, cytarabine; CNL, chronic neutrophilic leukemia; DAC, decitabine; DB: Döhle bodies; DRd, daratumumab-lenalidomide-dexamethasone; F, female; Hb, hemoglobin; HCT, hydrocortisone; HSCT, hematopoietic stem cell transplantation; Ig, immunoglobulin; KRd, carfilzomib-lenalidomide-dexamethasone; MGUS, monoclonal gammopathy of unknown significance; MCV, mean corpuscular volume; MTX, methotrexate; M, male; MM, multiple myeloma; Nc, not calculable; Nd, not detected; RBC, red blood cell; R-CHOP, rituximab-cyclophosphamide-doxorubicin-vincristin-prednison; TG, toxic granulations; US, ultrasound; VMP, bortezomib-melphalan-prednisone; VRD, bortezomib-lenalidomide-dexamethasone; VTD, bortezomib-thalidomide-dexamethasone; Y, years; YoS, Years of survival; /, not determined.

### Case 2

A 74-year-old man, known with IgA-type MGUS since 2003 and persistent neutrophilic leukocytosis since 2009, was diagnosed with spinal located diffuse large B-cell lymphoma (DLBCL) subtype ABC in 2013 (KI67 20%–25%, karyotype (46, XY[20]). Familial anamnesis did not include hemato-oncological pathologies. Medical therapy at presentation comprised nebivolol (2.5 mg QD) and spironolacton/altizide (12.5 mg/7.5 mg QD). Biochemical analysis showed leukocytosis of 43.69 × 10^9^/L (90.7% neutrophils) with M-spikes in both β- and γ-fractions.

Bone marrow biopsy revealed hypercellularity with increased representation of the myeloid lineage (mature and immature) and local clustering (5%–10%) of monotypic plasma cells (kappa-type, CD20-). Karyotyping resulted normal. Clonality between the spinal located DLBCL and bone marrow tissue was proved by polymerase chain reaction (PCR) (*IGH/IGK*).

Thus, the patient was diagnosed with bone marrow involved DLBCL, CNL, and MGUS. Six cycles of rituximab-cyclophosphamide-doxorubicin-vincristin-prednisone (R-CHOP), followed by rituximab monotherapy (administered twice), were given. In addition, intrathecal chemotherapy (methotrexate, cytarabine, and hydrocortisone) was administered three times. During therapy, bone marrow biopsy showed minimal CD5+ positivity (<0.1% all nucleated cells (ANC)) and isolated loss of the Y-chromosome (45,X,-Y[3]/46,XY[7]). *CSF3R* mutations were absent. Complete remission (CR) of DLBCL was obtained on PET-CT scan by September 2014. Asymptomatic neutrophilic leukocytosis remained during remission (>30 × 10^9^/L; >80% neutrophils).

In February 2016, an increasing absolute neutrophil count of 48.5 × 10^9^/L and M-spike were observed (IgA 13.5g/L). Urine kappa FLCs were not calculable. In June 2020, MGUS progressed to MM, type IgA kappa (R-ISS III, IgA 36.60 g/L); the total number of leukocytes normalized. Cytogenetic analysis showed hyperdiploidy with gain on chromosome 1q. *CSF3R*-analysis was not performed at this stage. Bone marrow biopsy showed hypercellularity and right-shifted myeloid cells with normal morphology. There was plasmocytosis of 55.7% ANC. The patient preferred no further therapies and deceased shortly after, roughly 7 years after CNL diagnosis.

### Case 3

In 2018, a 73-year-old woman was referred due to persistent leukocytosis for 1 year. Medical therapy comprised acetylsalicylic acid (80 mg QD), bisoprolol (2.5 mg QD), and rosuvastatin (10 mg QD). Besides significant weight loss (>10% within 12 months), no B-symptoms were present. Blood film analysis confirmed the presence of left-shifted neutrophilia with toxic granulations and Döhle bodies. Bone marrow biopsy showed hypercellularity (>95%) with myeloid hyperplasia, plasmacytosis of 10%–15% (lambda positive), and bone marrow fibrosis grade 1. Karyotyping resulted normal. NGS revealed mutated *ASXL1* (c.1934dup;p.Gly646Trpfs*12)(VAF 26%); *CSF3R* appeared normal (**Table 2**). The tentative diagnosis of CNL was made. No therapy was initiated.

**Table 2:** Genetic and molecular findings at diagnosis and follow-up.

	Patient 1 (F)			Patient 2 (M)			Patient 3 (F)		Patient 4 (M)		Patient 5 (F)	
	2012	2016	2018	2013	During R-CHOP (2014)	2020	Jul/18	Nov/18	May/19	Jun/20	Jul/19	Jan/20
**Stage of disease**	CNL+MGUS	CNL+MM	During follow-up	CNL+MGUS+DLBCL	CNL+MGUS+DLBCL	MM	CNL	CNL+MM	CNL+AML	Relapse AML	Smoldering MM	CNL+MM
**Karyotyping**	46,XX [10]	/	/	46,XY [3]	45,X,-Y [3] 46,XY [7]	/	46,XX [10]	46,XX,+5,+7,+9,+15	47,XY,-7,+14,+r,inc[2] 46,sl,-r[5] 46,sdl,del(12) (p11p13)[4]	46,XY,-7,del(12) (p11p13),+14[2] 46,XY, t(17;22) (q12;q12)[3] 46,XY[5]	46,XX[28]	46,XX [25]
**FISH**	/	Normal**	17p13/*TP53* ^+^ *IGH* ^-^	/	/	*IGH*/14q32^-^	/	*IGH*/14q32^+^ *FGFR*3 [t(4;14)]^-^ *MAF* [t(14;16)]^-^ **	/	/	Duplication 1q^+^ Deletion 16q23^+^ t(4;14)^-^ t(14;16)^-^ *TP53* ^-^ **	*BCR::ABL1* ^-^
**PCR/NGS**	*BCR::ABL1* ^-*^ *FLT3*-ITD^-^ *TP53* ^-^ V617F *JAK2* ^-^	*CSF3R* ^-^ *ASXL1* ^-^ V617F *JAK2* ^-^ *SETBP1* ^-^ *TP53* ^-^ ^***^	/	*IGH/IGK* ^+*^ *BCR-ABL1^-^* BCL2/*IGH* ^-^	*CSF3R* ^-^ *ASXL1* ^-^ V617F *JAK2* ^-^ *SETBP1* ^-^ *TP53* ^-^ ^***^	+1q	*ASXL1* ^+*^ (c.1934dup;p.Gly646Trpfs*12) *BCR-ABL^-^* *CSF3R* ^-^ V617F *JAK2* ^-^ *FLT3*-ITD^-^ *TP53* ^-^ ^***^	/	*CSF3R* ^+^ * (c.1853C>T; p.(Thr618Ile)) *SETBP1* ^+^ (c.2615T>C; p.(Ile871Thr)) *U2AF1^+^* (c.470A>C; p.(GLn157Pro)) *BCR::ABL1* ^-^ *ASXL1* ^-^ V617F *JAK2* ^-^ *FLT3*-ITD^-^ *TP53* ^-^ ***	/	*BCR::ABL1* ^-^ *CSF3R* ^-^ *AXSL1* ^*-*^ V617F *JAK2* ^-^ *FLT3*-ITD^-^ *TP53* ^-^ *IGH* ^-^ ***	*BCR::ABL1* ^-^ *CSF3R* ^-^ *AXSL1* ^*-*^ V617F *JAK2* ^-^ *FLT3*-ITD^-^ *TP53* ^-^ ***

*With “^-^”indicating the absence of a mutation; “^+^” indicating the presence of a mutation; **Cfr. Appendix 1 for the complete list of screened regions/loci by fluorescence *in situ* hybridization (FISH); ***Cfr. Appendix 2 for the complete list of screened genes by next-generation sequencing (NGS); “/” indicating “not determined”.

Four months later, the patient was diagnosed with lambda light chain MM (R-ISS I). Biochemical analysis revealed neutrophilic leukocytosis, hypogammaglobulinemia, and a normal β_2_-microglobulin concentration. Serum lambda FLCs were significantly increased (free λ: 276 mg/L (ref. 10–34) (**Table 1**)). There was significant proteinuria (6.79 g/24 h) with prominent lambda excretion (9816 mg/24 h). Bone marrow biopsy showed hypercellularity with segmented neutrophils (36.3% ANC) and increased central plasmocytosis (13.7% ANC) without blastosis. Congo Red staining resulted normal. Karyotyping revealed hyperdiploidy with gain on chromosomes 5, 7, 9, and 15. FISH detected IGH/14q32 rearrangement. Bortezomib-melphalan-prednisone (VMP) was initiated and halted after nine cycles as biochemical CR and minimal residual disease on PET-CT were obtained. Currently, more than 4 years after CNL and MM diagnosis, the patient is stable. Peripheral blood leukocyte count is <25 × 10^9^/L.

### Case 4

In 2019, a 69-year-old man was referred due to incremental neutrophilic leukocytosis and fatigue since several months. Medical history included diabetes mellitus type 2. Medical therapy comprised sitagliptin/metformin (50 mg/1000 mg BID), rosuvastatin (10 mg QD), and allopurinol (100 mg QD). Biochemical analysis showed normocytic anemia with concurrent neutrophilic leukocytosis and 2.7% blasts. Electrophoresis and immunofixation indicated MGUS (IgG lambda type). Bone marrow biopsy showed hypercellularity with blast excess (20%). Cytogenetic analysis identified a complex karyotype with monosomy of chromosome 7, trisomy of chromosome 14, and deletion of p12 (**Table 2**). *CSF3R* (44%), *SETBP1* (45%), and *U2AF1* (42%) appeared mutated. The diagnosis of AML secondary to CNL was made. Complete morphologic and cytogenetic remission was obtained after eight cycles of decitabine. Planned allogeneic HSCT was postponed due to flu and the SARS-CoV-2 pandemic. The patient relapsed several months later. Cytogenetic analysis detected the reoccurrence of previous described mutations in combination with a new translocation t(17;22) (**Table 2**). Despite that no CR was obtained after remission-reinduction, allogeneic HSCT was performed. Bone marrow analysis 2 and 4 months afterwards showed no blast excess; mutated *CSF3R* was absent. The patient recovered and is currently, more than 2 years after HSCT, in CR. Paraproteinemia and neutrophilic leukocytosis did not reoccur.

### Case 5

In 2020, a 66-year-old woman presented with recurrent headaches, fever and generalized myalgia/arthralgia since several months. Clinical investigation was unremarkable. Familial anamnesis did not include hemato-oncological abnormalities. There was no use of medicines. Medical history included successfully treated mammary carcinoma in 2010 (surgical resection, radio- and hormonal therapy; CR at presentation). In 2019, the patient developed auto-immune aortitis, successfully treated with corticoids. A few months later, the patient was diagnosed with smoldering MM (IgG lambda). Bone marrow biopsy then showed hypercellularity with a strong representation of the granulocytic lineage in intermediary and mature stages, toxic granulations, and sea-blue histiocytes. Central plasmocytosis was 10.5%. No central blast excess was observed. Cytogenetic and molecular analysis resulted normal. Since the diagnosis of smoldering MM, there was persistent neutrophilia. Given the suspicion of CNL, the patient was shortly treated with hydroxycarbamide, which was rapidly stopped due to provoked cytopenia. Relapse of breast carcinoma was excluded and PET-CT resulted normal.

A few months later, smoldering MM evolved into MM (R-ISS II). Treatment with bortezomib-lenalidomide and dexamethasone (VRD) was initiated and resulted in partial response (PR). The patient refused intensification with autologous HSCT. VRD was stopped after three cycles due to neurological complications. According to patient requirement, low-dose lenalidomide (5 mg) was continued in monotherapy and PR was maintained. Under lenalidomide, the patient progressively developed nephrotic syndrome. Renal biopsy showed glomerulonephritis with mesangial IgA deposits, probably not related to the hematologic condition. Currently, the patient is in follow-up; leukocyte count remains normal.

## Methods

Five patients diagnosed with CNL between 2012 and 2020 were included in this study. In retrospect, CNL diagnosis was based on the 2016 WHO diagnostic criteria (4). Cytogenetic and molecular analysis of patients 1–3 was performed at the University Hospitals Leuven, Belgium. Cytogenetic analysis of patient 4 was performed at *Université Catholique de Louvain*, CHU UCL Namur, Belgium. Molecular analysis of patient 4 was performed at the University Hospitals Leuven. Cytogenetic analysis of patient 5 was performed at *Université Catholique de Louvain Saint-Luc*, Woluwe-Saint-Lambert, Belgium. Molecular analysis of patient 5 was performed at *Institut de Pathologie et de Génétique* (IPG), Charleroi, Belgium. We report cytogenetic and molecular analyses at diagnosis and during follow-up. Used probes and screened genes by FISH and NGS are clarified in the appendices. Patients 1–3 are treated at the University Hospitals Leuven. Patient 4 is treated at Université Catholique de Louvain, CHU UCL Namur, and patient 5, at Université Catholique de Louvain Saint-Luc, Woluwe-Saint-Lambert.

## Results

In this series the median age of CNL diagnosis is 69 years, 40% of patients (n = 2) are men; 20% (n = 1) of patients carried mutated *CSF3R.* B-symptoms and splenomegaly were present in 40% (n = 2) and 20% (n = 1) of patients, respectively. Three patients (66%) showed a predominance of lambda light chain expression. Four patients (80%) evolved to multiple myeloma. Hydroxycarbamide was shortly initiated in one patient. All patients received treatments focusing on associated malignancies such as AML and MM, among these 40% (n = 2) underwent HSCT. Two patients died after a median time of survival of 8 years after CNL diagnosis. Three patients (66%) are currently in follow-up (approximately 3 years after diagnosis) and show no signs of reoccurring leukocytosis.

## Discussion and review of the literature

### CNL laboratory features and epidemiology

In 1920, CNL was first described as “polymorphonuclear neutrophil hyperleukocytosis” (9). Roughly 200 CNL cases are currently reported in literature; however, many cases may not meet the WHO-defined diagnostic criteria (1, 3). The lack of cytogenetic markers has complicated accurate diagnosis in the past. Recent identification of oncogenic driver mutations in *CSF3R* resulted in the updated WHO diagnostic criteria in 2016 (1, 4, 5).

Besides the presence of mutated *CSF3R*, the diagnostic criteria include a peripheral blood leukocytosis of ≥25 × 10^9^/L with ≥80% neutrophils (segmented and band), <10% peripheral neutrophil precursors, absence of monocytosis/eosinophilia/basophilia, and rarely observed peripheral myeloblasts. Arber et al. provide a complete list of the current diagnostic criteria (4, 7, 10). Levels of vitamin B12 and lactate dehydrogenase (LDH) are frequently elevated but are no solid criteria. Neutrophil morphology typically presents with non-specific characteristics such as toxic granulations and Döhle bodies (8, 11). Bone marrow biopsy shows hypercellularity with increased numbers of normal maturating neutrophilic granulocytes. Chronic myeloid leukemia and other *BCR::ABL1* negative MPNs must be excluded; rearrangement of *PDGFRA, PDGFRB, FGR*, or *PCM1::JAK2* should be absent. Without mutated *CSF3R*, the diagnosis is possible if persistent non-reactive neutrophilia (≥3 months) is present. In this context, demonstrated myeloid clonality by cytogenetic or molecular analysis is preferred (4, 10).

When strictly applied, only cases 1–3 and 5 meet the CNL criteria concerning leukocytosis. In case 4, even before the diagnosis of AML, peripheral blood leukocytosis was <25 × 10^9^/L with <80% neutrophils. However, the persistence of leukocytosis (>3 months) without an identifiable cause of reactive neutrophilia combined with mutated *CSF3R* strongly supports the CNL diagnosis. In AML, the incidence of mutated *CSF3R* is approximately 1%. This case emphasizes the risk of an arbitrary leukocyte number as an absolute criterion for diagnosis (5, 12).

Elliott et al., analyzing 40 WHO-defined CNL cases, reported a median age of 66 years (range 16–86). The majority of these cases were men (56%) (3). In our series two of five patients (40%) were male and the median age of diagnosis was 69 years. Splenomegaly, which is removed as a solid criterion in the current WHO criteria, was present in one of five (20%).

### Mutated *CSF3R* as key driver in CNL

*CSF3R* is the gene coding for the receptor of colony-stimulating-factor-3 (CSF3), a primary neutrophil growth factor. Mutated *CSF3R* is considered as a solid CNL-defining criterion. In CNL, the mutational frequency of *CSF3R* is approximately 60%–80%, with *CSF3R*T618I as the most frequent reported mutation (7, 13). *CSF3R*T618I occurs in the extracellular or transmembrane domain of the receptor and results in the activation of the JAK-STAT pathway; which explains the marked clinical improvement after the initiation of ruxolitinib in some patients. Other mutations preferentially activate the SRC tyrosine kinase. We refer to Maxson et al. for qualitative information about *CSF3R (* 5, 6, 13). In MG-associated CNL, the incidence of mutated *CSF3R* appears to be significantly lower; however, data is scarce (7).

### CNL and plasma cell disorders

According to the WHO diagnostic criteria, plasma cell neoplasms should be excluded in the absence of mutated *CSF3R (* 4). Nevertheless, up to 32% of CNL cases report concurrent plasma cell dyscrasias, with the predominance of lambda light chain expression (66% or 3/5 in our series) (1). All patients showed sign of MG at the time of CNL diagnosis; 80% (4/5) eventually evolved into MM. Larger data sets are necessary to evaluate whether patients with CNL-associated MGUS carry an inherent higher risk to evolve into MM.

It is unclear whether MG-associated CNL has to be considered as a genuine myeloproliferative neoplasm or neutrophilic reaction, for example, secondary to production of plasma cell-derived cytokines such as G-CSF (1). Quantification of G-CSF serum concentration was not performed in our series but could be useful. The lower frequency of mutated *CSF3R*, combined with the longer median time of survival in MG-associated CNL, may indicate different etiopathogenesis (1, 7). Only patient 4 carried mutated *CSF3R*. MG-associated CNL shows a median time of survival of approximately 5 years, compared to 24 months in true CNL (3, 7, 14). Patients 1 and 2 show a median time of survival of 8 years after CNL diagnosis. Patients 3, 4, and 5 are in follow-up, roughly 3 years after CNL diagnosis.

Some authors stress the few reported chromosomal abnormalities and better survival as evidence for a provoked neutrophilic reaction in MG-associated CNL (14, 15). However, Nedeljkovic et al. reported the presence of a homozygous *JAK2*V617F mutation in a patient with CNL associated with plasma cell myeloma, demonstrating molecular evidence of clonality in the absence of mutated *CSF3R (* 16). Mutated *ASXL1* in patient 3 at the time of CNL diagnosis is a sign of clonality in the absence of mutated *CSF3R*.

### Cytogenetic abnormalities

#### Karyotype

Y-chromosome loss, as in patient 2, is frequently reported in patients with hematological disorders. It remains unknown whether Y-chromosome loss should be considered as an age-related phenomenon or cytogenetic marker for malignancy (17). Copy number alterations, resulting in gain of chromosomes such as +5, +7, +9, and +15 in patient 3, are one of the most prominent perturbations in MM. The gain on chromosomes 9 and 15 may play an important role in MGUS transformation (18). Abnormalities of chromosome 1 are observed in approximately 25% of patients at MM diagnosis. The gain on 1q, as in patients 2 and 5, is associated with inferior survival (19).

At CNL diagnosis, patient 4 carried monosomy 7, trisomy 14 and 12p-deletion. This latter is associated with progression to post-MPN AML (20). Trisomy 14 is a rare cytogenetic abnormality in myeloid neoplasms such as AML. Isolated trisomy 14 is indicated as an early event in leukemogenesis; however, more research on its clinicopathological features is needed. Monosomy 7 is frequently reported in myeloid malignancies and is detected in previous CNL-cases who developed secondary AML (3, 21, 22). The translocation t(17;22)(q12;q12), which occurred in patient 4 during AML relapse, is associated with myelodysplastic syndrome (MDS) (23).

#### Additional mutations

Specific mutations may indicate a negative prognosis in CNL, even if, by our knowledge, currently no mutations are validated as prognostic markers. Mutated *ASXL1* as in patient 3, resulting in disrupted epigenetic regulation, is associated with a negative prognosis in CNL. Mutational frequency is varying from 30% to 81% (1, 22).

Mutated *TP53* is characterized as a negative prognostic factor in myeloid and lymphoid malignancies (24, 25). Monoallelic loss of *TP53* was detected in patient 1 during follow-up; the patient deceased 2 years afterwards. The relevance of mutated *TP53* in MG-associated CNL is unclear.

Rearrangements involving the *IGH locus* on chromosome 14q32 frequently occur in MM and are associated with standard risk (26). Translocations in 14q32 less frequently co-occur with hyperdiploid chromosomes such as in patient 3 (27).

*SETBP1*, considered as a driver oncogene, is mutated in approximately 33% of CNL cases (7). It is associated with poor prognosis in various myeloproliferative phenotypes, including secondary AML as in patient 4. Whether mutated *SETBP1* is associated with poor survival in CNL is unknown (22, 28).

A limited number of cases report the presence of *JAK2*V617F mutations in CNL; little is known about its true prevalence in WHO-defined CNL cases (7, 29–34). *JAK2*V617F and *CSF3R*T618I appear to be mutually exclusive (7, 10, 35). None of the patients in our series carried *JAK2*V617F.

Mutated spliceosome-associated genes such as *SRSF2* and *U2AF1* have been reported in CNL. Meggendorfer et al. report a *SRSF2* mutational frequency of 21% (4/14 cases); others report a frequency of 0% (0/10) (35–37). Patient 4 carried the Glutamin(Gln)157pro mutation in the *U2AF1* gene at the moment of CNL and AML diagnosis. Mutated *U2AF1* results in predisposition to AML (38, 39). Other CNL-associated mutations, such as *TET2* and *RUNX1*, were absent in our cases. *TET2* mutations have an estimated mutational frequency of 29% in CNL (1, 36). Previous authors postulated that cooperating *RUNX1* and *CSF3R* mutations in CNL may result in disease progression, resistance to ruxolitnib and may act as an early marker of AML transformation (40, 41).

### Management

#### CNL

Currently, no standard of care management exists for CNL. Historically, splenic irradiation and splenectomy were performed to reduce tumor bulk and abdominal discomfort. Nevertheless, splenectomy is associated with worsening of neutrophilia in CNL. The only potentially curative treatment is HSCT (1). Allogeneic HSCT showed a one-year overall survival rate of 40% in patients with CNL. However, data regarding clinical outcomes and the most optimal regimens of HSCT are scarce (3, 10, 42, 43). In our series, autologous and allogeneic HSCTs were performed in patient 1 due to progressive MM and in patient 4 due to AML, not CNL specifically.

The use of “7+3” induction chemotherapy in CNL has not been able to induce hematological remission. One report describes a young patient in the blast phase who attained a second chronic phase following induction chemotherapy (anthracycline and cytarabine) (44).

Cytoreductive agents, such as hydroxyurea and interferon-alpha (IFN-α), have demonstrated efficacy in controlling leukocytosis and splenomegaly. Currently, hydroxyurea is the most frequently used first-line agent; nonetheless, 25% of patients appear to be refractory (3, 35). Few reports mention durable remission and good tolerability after IFN-α initiation (45–47). No IFN-α is used in our series as it is not reimbursed in our country.

Promising targeted therapies are being investigated since the identification of mutated *CSF3R*. Depending on the mutation downstream signaling pathways through JAK-STAT or SRC tyrosine kinase are activated, these may be inhibited by ruxolitinib and dasatinib, respectively (5, 10, 42). An overall response rate of 32% was observed in patients with *CSF3R*-mutated CNL treated with ruxolitinib; patients harboring *CSF3R*T618I were most likely to respond (48). None of these agents were used in our series.

#### MG-associated CNL

As in CNL, there is no standard of care management for MG-associated CNL (1). No specific anti-myeloma regimen can be recommended, but conventional chemotherapy, immunomodulatory drugs, proteasome inhibitors, and anti-CD38 monoclonal antibodies may reduce neutrophil counts to a variable extent. However, it is difficult to discriminate a direct effect on the CNL clone from an indirect reduction by the suppression of the malignant plasma cell clone. Keeping in mind the more favorable prognosis of MG-associated CNL, treatment strategies in our series were primarily focused on associated plasma cell dyscrasias and AML.

In patient 1, autologous HSCT was performed after VTD therapy. Subsequently, the patient received therapy existing out of KRd, DRd, and bendamustine due to MM progression. No reoccurrence of neutrophilia was observed after the initiation of VTD and following therapies. However, as various therapies were administered in a short time frame, no conclusion on the effectivity of individual therapies in CNL can be made. As mentioned, HSCT in patient 1 and 4 was performed due to progressive MM and AML respectively, not CNL specifically.

Decitabine, a demethylating agent, was initiated in patient 4 and resulted in complete morphologic and cytogenetic remission after eight cycles. Previous authors reported complete remission and suggested decitabine as a potential effective therapeutic agent in patients with secondary AML; nonetheless, data is scarce (49).

Bortezomib, a proteasome inhibitor, was administered in patients 1, 3, and 5 for the treatment of MM. To our knowledge, only one previous case report describes the use of bortezomib in CNL-MM, resulting in the complete resolution of both leukocytosis and MG (50). Reduced neutrophil count is a common observation in the use of bortezomib. Bortezomib could act directly through the effect on proteasomes in neutrophils, or indirectly through its influence on cytokine concentration. The administration of bortezomib in patients 1 and 3 resulted in a VGPR, while in patient 5 a partial response was achieved. Data of immunomodulatory drugs in MG-associated CNL, such as thalidomide and lenalidomide in patients 1 and 5 of our series, are scarce. The number of leukocytes normalized in all of these patients.

## Conclusion

Chronic neutrophilic leukemia is a rare but potentially aggressive myeloproliferative neoplasm with a median survival of approximately 24 months. A non-negligible number of CNL cases are diagnosed with plasma cell dyscrasias; these patients show a more favorable prognosis with a median survival of 5 years. The discovery of oncogenic *CSF3R* driver mutations raised the diagnostic accuracy of CNL and resulted in the updated WHO diagnostic criteria in 2016. *CSF3R* tends to be less frequently mutated in CNL associated with monoclonal gammopathies (MG), challenging the diagnosis. Better survival and lower *CSF3R* mutational frequency may suggest a different etiopathogenesis between pure CNL and MG-associated CNL. Owing to the rarity of CNL, there is no defined standard of care. Currently, hematopoietic stem cell transplantation (HSCT) is the only curative treatment. This series provides an overview of cytogenetic evolution and treatment in MG-associated CNL. More knowledge about occurring mutations and the order of acquisition will hopefully result in better therapeutic approaches and outcomes.

## Data availability statement

The raw data supporting the conclusions of this article will be made available by the authors, without undue reservation.

## Ethics statement

Written informed consent was obtained from the individual(s) for the publication of any potentially identifiable images or data included in this article.

## Author contributions

GV: data curation (equal), formal analysis (equal), visualization (equal), writing-original draft (lead), writing-review and editing (supporting). MD: data curation (equal), formal analysis (equal), visualization (equal), writing-original draft (supporting), writingreview and editing (supporting). VH: data curation (equal), formal analysis (equal), visualization (equal), writing-original draft (supporting), writing-review and editing (supporting). CG: data curation (equal), formal analysis (equal), visualization (equal), writingoriginal draft (supporting), writing-review and editing (supporting). LM: data curation (equal), formal analysis (equal), visualization (equal), writing-original draft (supporting), writing-review and editing (supporting). TD: data curation (equal), formal analysis (equal), visualization (equal), writing-original draft (lead), writing-review and editing (lead).

## Appendix 1

*A. List of screened regions/genes by Fluorescent In Situ Hybridization (FISH) in patient 1*

LSI *CDKN2A*(9p21)(SO)/*CEP9(SG*) [9p21/9p11.1-9q11.1, Abbott], LSI *TP53*(SO)/*CEP17*(SG) [17p13/17p11.1-17q11.1, Abbott], LSI *IGH*(DC BA) [14q32, Abbott], *CEP9*(SO) [9p11.1-q11.1, Abbott], *CKS1B*(SG)/*CEP1*(SO) [1q21/1p11.1-1q11.1, CME + Abbott]

B. *List of screened regions/genes by Fluorescent In Situ Hybridization (FISH) in patient 2*

XL *IGH*(DC BA) [14q32, Metasystems]

C. *List of screened regions/genes by Fluorescent In Situ Hybridization (FISH) in patient 3*

LSI *IGH*(DC BA) [14q32, Vysis], LSI *IGH*(SG)/*CMAF*(SO)(DC DF) [14q32/16q23, Vysis], LSI *IGH*(SG)/*CCND1*-XT(SO) (DC DF) [14q32/11q13, Vysis], LSI *IGH*(SG)/*FGFR3*(SO) (DC DF) [14q32/4p16, Vysis]

D. *List of screened regions/genes by Fluorescent In Situ Hybridization (FISH) in patient 5*

LSI 4q12 (*FIP1L1*-PDGFRA) [Vysis tri-color rearrangement probe], LSI *PDGFRB* [Vysis], LSI *JAK2* [Kreatech], LSI *FGFR1* [Zytovision], LSI *BCR*/ABL(ES) [9q34.1/22q11.23, Vysis], *TPI *17p13.1/*CEP17*(D17Z1) [Metasystems], *IGH/FGFR3* (DC DF) [14q32/4p16, Metasystems], *IGH/MAF* (DC DF) [14q32/16q23, Metasystems]

## Appendix 2

*A. List of screened genes by Next-Generation Sequencing (NGS) in patient 1,2, 3 & 4.*

*ABL1, ASXL1, ATRX, BCOR, BCORL1, BRAF, CALR, CBL, CBLB, CBLC, CDKN2A, *CSF3R*, CUX1, DNMT3A, ETV6, EZH2, FBXW7, FLT3, GATA1, GATA2, GNAS, IDH1, IDH2, IKZF1, JAK2, KMD6A, KIT, KRAS, MPL, MYD88, NOTCH1, NPM1, NRAS, PDGFRA, PHF6, PTEN, PTPN11, RAD21, RUNX1, SETBP1, SF3B1, SMC1A, SMC3, SRSF2, STAG2, TET2, TP53, U2AF1, WT1, ZRSR2*

B. *List of screened genes by Next-Generation Sequencing (NGS) in patient 5*

*ASXL1, CEBPA, DNMT3A, FLT3, IDH1, IDH2, cKIT, NPM1, RUNX1, TET2, TP53, WT1, SF3B1, SRSF2, U2AF1, JAK2, MPL, CALR, EZH2, SETBP1, *CSF3R**
